# A case of a 55-year-old female with uterine prolapse grade IV: a rare clinical image

**DOI:** 10.11604/pamj.2023.46.10.41360

**Published:** 2023-09-08

**Authors:** Switi Jawade, Kavita Gomase

**Affiliations:** 1 Department of Obstetrics and Gynaecology Nursing, Smt. Radhikabai Meghe Memorial College of Nursing, Datta Meghe Institute of Higher Education and Research (DU) Sawangi Meghe Wardha, Wardha, Maharashtra, India

**Keywords:** Uterine prolapse, hysterectomy, hydronephrosis, clinical image

## Image in medicine

In the female reproductive system anatomical abnormality is uterine prolapse and it developed when uterus dropped into the vagina. It may cause obstructive uropathy with unilateral or bilateral mild hydronephrosis. The more risk for women who increasing aged and decreasing oestrogen level and it can diagnose by pelvic examination. Treatment for prolapse is severe surgical treatment include uterine suspension or hysterectomy. Here we reported patient came to the hospital with chief complaint of something coming out of her vagina, it was progressive in nature and progressed to current size, increase frequency urgency, frequent micturition (5 to 6 time/day), backache. There is significant past history of hypertension since 2 years and she is taking Tab. Amlo 5 mg BD. On the pelvic and physical examination and ultrasound sonography abdomen and pelvis done that revealed grade IV hydro-uretero nephrosis right kidney: 12.5x6 cm and left kidney: 10x8.5 it shows severe dilatation of right renal pelvic congestion syndrome and liver calcific focus of size 2.3x2.7 mm present in segment 4 of left lobe. After diagnosing uterine prolapse grade IV, patient underwent surgical treatment of vaginal hysterectomy.

**Figure 1:**
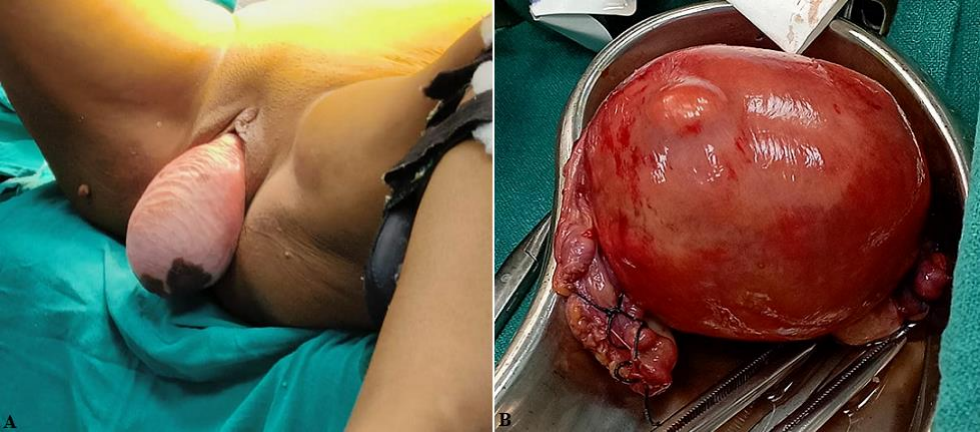
A) total uterine prolapse associated with bilateral hydronephrosis; B) total prolapse uterus

